# Trends in mathematical modeling of host–pathogen interactions

**DOI:** 10.1007/s00018-019-03382-0

**Published:** 2019-11-27

**Authors:** Jan Ewald, Patricia Sieber, Ravindra Garde, Stefan N. Lang, Stefan Schuster, Bashar Ibrahim

**Affiliations:** 1 Matthias Schleiden Institute, Bioinformatics, Friedrich Schiller University Jena, Ernst-Abbe-Platz 2, 07743 Jena, Germany; 2 Max Planck Institute for Chemical Ecology, Hans-Knöll-Str. 8, 07745 Jena, Germany; 3 Centre for Applied Mathematics and Bioinformatics, Gulf University for Science and Technology, 32093 Hawally, Kuwait

**Keywords:** Dynamic optimization, Game theory, Agent-based modeling, Equation-based modeling, Host–pathogen interactions, Crypsis

## Abstract

Pathogenic microorganisms entail enormous problems for humans, livestock, and crop plants. A better understanding of the different infection strategies of the pathogens enables us to derive optimal treatments to mitigate infectious diseases or develop vaccinations preventing the occurrence of infections altogether. In this review, we highlight the current trends in mathematical modeling approaches and related methods used for understanding host–pathogen interactions. Since these interactions can be described on vastly different temporal and spatial scales as well as abstraction levels, a variety of computational and mathematical approaches are presented. Particular emphasis is placed on dynamic optimization, game theory, and spatial modeling, as they are attracting more and more interest in systems biology. Furthermore, these approaches are often combined to illuminate the complexities of the interactions between pathogens and their host. We also discuss the phenomena of molecular mimicry and crypsis as well as the interplay between defense and counter defense. As a conclusion, we provide an overview of method characteristics to assist non-experts in their decision for modeling approaches and interdisciplinary understanding.

## Introduction

Pathogenic organisms have been an immense burden since the beginning of civilization and still continue to afflict us through deadly infectious diseases that affect humans, animals, or plants. Even today, in a world where we have developed advanced antibiotics designed to specifically target and suppress pathogens, microbial pathogens continue to be a leading cause of disease that causes major loss of human lives, crops, and livestock. For example, human infections are estimated to cause over 8 million deaths in 2019 [90] and plant pathogens are responsible for a 20–30 % yield loss of major food crops [108]. While many infectious diseases have practically been eradicated, new problems such as antibiotics resistance have emerged, which considerably increase the clinical and economical burden [131]. Besides bacteria and viruses, pathogenic fungi such as *Candida albicans* or *Aspergillus fumigatus* are an underestimated threat [18].

In conjunction with experimental and clinical investigations, computational and mathematical approaches have turned out to be highly valuable in understanding and diagnosing host–pathogen interactions (HPI) and devising optimal therapies [9, 30, 109]. Furthermore, computational modeling of biological conditions can save time and money compared to wet lab experiments, and can simulate certain processes that are hardly realizable in experiment [21].

Computational approaches have led to several success stories already. They have been very helpful in predicting, assessing, and controlling epidemics [117]. To that end, epidemiological models are used. On the micro host–pathogen level considered in this review, for example, the metabolism of *Trypanosoma brucei*, the causative agent of the sleeping disease, was analyzed by mathematical modeling. That led to the prediction that glucose transport is a promising drug target without collateral damage to the host [3], as has indeed been confirmed in experiment [51].

In the present review, we give an overview of computational approaches describing HPI, with a special reference to dynamic optimization, evolutionary game theory, and the modeling of spatial phenomena. The following aspects are covered, in this order, in the sections “Dynamics of infection and optimization”, “Costs and benefits”, “Molecular mimicry and crypsis”, “Spatial properties”, and “Defense, counter defense and counter-counter defense”: the dynamics of infection, costs, and benefits of HPI as described by game theory, molecular crypsis of pathogens, spatial properties, and the levels of defense interactions. As the name suggests, dynamic optimization is based on optimality principles [38], which are in line with Darwin’s concept of ’survival of the fittest’. Also game theory is based on that concept, the idea being that each ’player’ evolves such as to maximize its fitness [57, 59]. However, as the counterparts in the game interfere, they may hinder each other to attain optimal states.

The question arises why various computational methods are applied. In some cases, simple optimization (rather than game theory) is sufficient, notably if the tendencies of the ’players’ (e.g., organisms) to increase their fitness are not in conflict with each other. Many processes can be described by alternative methods. For example, the interplay of alleles can often be described both by population genetics and game theory [60]. It depends on the aim of study which method is most suitable [26] or may be, to some extent, a matter of taste. This will be discussed in more detail in the section “Conclusion and outlook”.

## Dynamics of infection and optimization

**Fig. 1:**
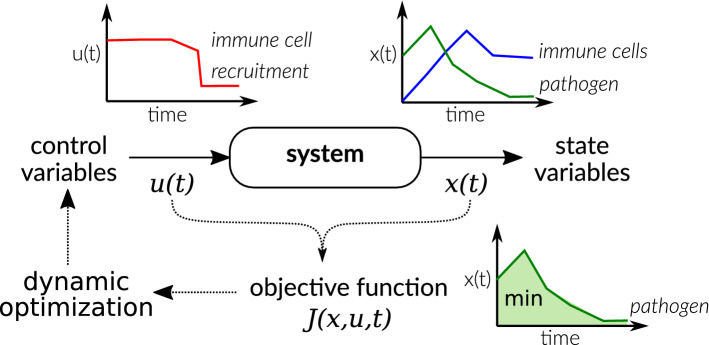
Basic concept of dynamic optimization illustrated by a simple host–pathogen system. A system, in the depicted example the growth of pathogens (green) and their phagocytosis by immune cells (blue), is described by state variables [*x*(*t*)]. The behavior of the system is influenced by control variables [*u*(*t*)], for example the recruitment of immune cells (red) to combat the pathogen. The control variable is optimized with regard to an objective function to find, for example, the optimal time course of recruitment of immune cells to minimize the pathogen load. Such time courses often show a switch-like behavior between upper and lower bounds (the so-called bang–bang control)

During the interaction of host and pathogens, speed and timing are crucial for both to survive. Therefore, the method of dynamic optimization is valuable to describe and understand infection processes. Originating from engineering, dynamic optimization, also called optimal control (closed-loop problems), describes biological systems by an ordinary differential equation (ODE) system whose behavior is influenced by control or decision variables [5, 80]. On a molecular level, host and pathogenic cells, for example, use enzyme levels (as changed by gene expression, etc.) to control the flux in metabolic pathways and respond to environmental changes [37, 38, 73]. In models of HPI, often cells and their behaviors like proliferation or recruitment are viewed as control variables. These control variables are then optimized with regard to an objective function to get a time-optimal strategy controlling the behavior of the biological system (see Fig. 1). While there are many models of infections and HPI using ODEs [35], dynamic optimization is less frequently used to study host–pathogen systems.

Commonly, dynamic optimization is used for the analysis of transmission dynamics and epidemiology by extending the popular Susceptible-Infected-Recovered models [35, 113]. Although these models are valuable to obtain strategies controlling the spread of infections, we want to emphasize, in this review, dynamic optimization models describing infection processes and HPI within the host.

Dynamic optimization problems focusing on within-host dynamics are formulated in two main fields of application. In the first field, parts of the immune response itself are described by control variables. Defense strategies against pathogens are determined which maximize the health of the host and minimize the number of pathogens (see the example in Fig. 1). This idea was applied already more than 40 years ago by Perelson and colleagues in a series of publications studying the optimization of proliferation and differentiation of lymphocytes [98–100]. Later, Shudo and Iwasa postulated two dynamic optimization problems for these processes. First, they investigated the dynamic optimization of different defense options of the host, which differ in their ability to inhibit the growth of the pathogen, damage to host cells, or their time delay [115]. In a subsequent work, the immune response was viewed as an optimizing problem where the number and proliferation of immune cells is optimized while controlling pathogenic cells and minimizing tissue damage by immune cells [116].

Recently, Dühring et al. [29] applied dynamic optimization to the question of macrophage replication during their interaction with *C. albicans* and could provide evidence that immediate phagocytosis of fungal cells is mostly preferred over replication to increase immune cell number first. This supports the recommendation ’hit hard and hit fast’ of Paul Ehrlich stated a hundred years ago about the combat against microbes [34]. This characteristic is disclosed by nearly all dynamic optimization models of the immune response and is generically illustrated in Fig. 1. It shows that pathogens are best controlled by a quick and strong response. The increase in immune cells is then due to (fast) recruitment rather than (slow) proliferation. Mathematically, this can be easily explained by the non-linear exponential growth of pathogens. Due to this fast growth, also the effort to clear the pathogen would rise exponentially with time if applied later.

Empirically, the strategy ‘hit hard and hit fast’ has been proven to be of advantage for several reasons. First, a high average steady-state concentration of antimicrobial drugs enables a shorter antibiotic treatment and prevents generation of resistant strains [84]. Second, antibiotics are administered with a higher initial dose, the so-called loading dose. The loading dose is given to rapidly achieve an effective drug concentration in the blood and tissues, and underlines the importance of a quick response [86]. Additionally, a fast and strong response reduces peak microbial load and, therefore, lowers the risk of a septic shock [76].

Similar ideas are investigated in the second main field of models using dynamic optimization. Here, Stengel et al. [121, 122] introduced dynamic models to determine time-optimal medical treatment strategies for an arbitrary pathogen. In that and subsequent work, the idea is to determine a time-optimal treatment schedule of using antibiotics, antivirals, etc., and also the optimal choice of different options, notably antimicrobials, healing factors, or immune enhancements [120–122].

Studies determining the time-optimal dosing strategies can help to reduce host damage, e.g., by sparing the commensal microbiome during the antibiotic treatment [97], while keeping a fast and strong intervention against pathogens. More recently, the modulation of inflammation during infection was investigated by multiple authors, who determined optimal dosing schedules of mediators for pro- or anti-inflammation [6, 24, 138].

All of the models discussed above focus on the optimization of the host’s health status. However, dynamic optimization can also be applied to understand the evolution of virulence as shown by Ebert and Weisser [31], who determined the optimal time point to kill the host from the viewpoint of a parasite.

In addition to the stand-alone models, dynamic optimization has emerged as a valuable approach in multi-scale modeling to complement other models with different scale or scope [109]. An example is the model of Chen et al. [20], which extends the idea of optimal treatment scheduling proposed by Stengel et al. [121]. This leads to a dynamic game-theoretical model. That work as well as the above-mentioned publication on the dynamics of macrophage replication and *C. albicans* evasion strategies [29] show that the combination of dynamic optimization and game theory is valuable to gain insights into the dynamics and evolutionary aspects of HPI. Moreover, aggregation of dynamic optimization with agent-based models describing the control of population dynamics in a 2D (e.g., on a lung epithelium) or 3D environment illustrates the potential for multi-scale models of infections using both approaches [2, 39].

Despite the presented successful application of dynamic optimization to HPI, an even closer integration with experimental data as well as more concrete suggestions for clinical applications are worthwhile. A similar point was made by Eftimie et al. [33], highlighting optimal control as a valuable mathematical approach in immunology, which can improve clinical intervention strategies. A role model is the application of optimal control to the management of diabetes, where dose regime optimization is realized not only in silico but also in clinical trials [43, 83].

## Costs and benefits

In the evolution of HPI, the costs and benefits of the employed strategies determine the fitness of both host and pathogen. As a mathematical description, game theory [46, 57] has proven to be of great value, and describes the difference between benefits and costs of strategies in a payoff matrix [11, 59, 127]. Based on this, stable solutions (sets of strategies) can be calculated, which are called Nash equilibria and are predictions for evolutionarily favorable strategies. The generalization of that concept is called evolutionarily stable strategy (ESS) [46, 57]. Game theory can provide insights into the evolution of interaction phenomena like the persistence of a pathogen. Nevertheless, most models are small and comprehensible, and the solutions are easily obtainable. While the payoffs are often difficult to quantify exactly, it is often sufficient to define the order relations between them to determine the Nash equilibria. A drawback of the approach is that the high level of abstraction can hamper a direct experimental validation.

In the following, we summarize different types of games and show their applications to HPIs. Typically, host and pathogens are considered as players with different strategies and unequal payoffs resulting in asymmetric games (see Table 1). Most models in game theory consider two possible strategies for each player. Accordingly, many game-theoretical models describing HPI are asymmetric two-player two-strategy games.

Intuitive strategies for host and pathogenic cells are either aggressive or non-aggressive against the other cell type. This is also referred to as attack and silence or as ’killer’ and ’diplomat’, respectively (see Tables 1 and 2). As one of the first game-theoretical models of HPI, Renaud and de Meeüs [106] found that there is always the ESS in which pathogen and host are aggressive (attack against host and suppression of pathogen, respectively). Only for some parameter values, the ’diplomat’ strategy is a solution as well. That is, ’war’ is always a stable situation, while ’peace’ is an additional stable situation (implying bistability) only under certain conditions, notably if $T_\text {P} < R_\text {P}$ and $S_\text {H} < R_\text {H}$ in Table 2 (notation according to Prisoner’s Dilemma: *R*, Reward for mutual cooperation; *T*, Temptation to defect; *S*, Sucker’s payoff; *P*, Punishment for mutual defection). This implies that the cost of virulence (pathogen) and the cost of resistance (host) exceed the loss due to hosting the pathogen.

**Table 1:**
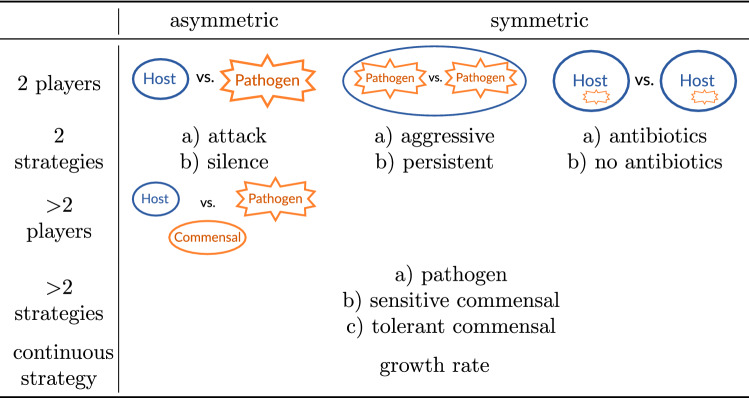
Types of host–pathogen games

Other asymmetric models focus on intracellular and persistent bacteria and gain insights into evolutionary advantages of attenuated virulence [124] or localization [36] as strategies of pathogenic bacteria. The opportunistic fungal pathogen *C. albicans* aroused attention due to its medical relevance and morphological switch from yeast-like to filamentous growth during infection. Interestingly, this was first studied by symmetric games (which are easier to analyze) between fungal cells, with the immune cells being considered as a constant environment [61, 128] (see Table 1). More recent studies, however, describe asymmetric games between host and pathogenic fungal cells and focus on certain experimental aspects like expression of transcription factors [127] for filamentous growth or evasion strategies resulting in non-lytic expulsion [29].

**Table 2:**
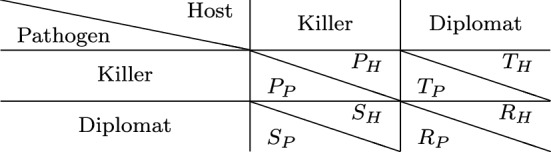
Example of a general payoff matrix for games between host and pathogen cells. Adapted from [106] with notation according to Prisoner’s Dilemma: *R* Reward for mutual cooperation, *T* Temptation to defect, *S* Sucker’s payoff, and *P* Punishment for mutual defection

In addition, symmetric games of pathogens competing within a host are described by continuous strategies and enable Nash equilibria to be calculated and predictions for optimal growth rates [17] or toxin production of pathogens [95] to be made. The latter is also described as an N-player game with two strategies for producing a host cell wall toxin or to cheat and results in a Volunteer’s Dilemma [95]. On the other side, games between hosts facing pathogens can also be modeled by N-player games. For example, some models describe the overuse of antibiotics resulting in a Tragedy of the Commons [41, 105]. This can be used to draw conclusions for the prescription of antibiotics.

To better explain the biodiversity of pathogens or the host’s microbiome, models with three players and/or three strategies are defined. For example, Wu and Ross [135] investigated the asymmetric game of two types of commensals (sensitive or non-sensitive to antibiotics) and a pathogenic bacterial species. That model explains the common observation that supporting commensal bacteria can alone be effective to eradicate pathogenic bacteria in the gut of a host. Another interesting perspective was investigated by Morozov and Best [87], who used game theory to model the interplay of predators, prey, and pathogens infecting the prey.

Furthermore, an emerging trend in modeling HPI is to extend and represent game-theoretical models with graphs and lattices adding spatial and stochastic characteristics. With these multi-scale models using methods of agent-based modeling, the invasion of bacteria [72] and lung infections by *A. fumigatus* have been modeled [104].

## Molecular mimicry and crypsis

An important aspect in HPI is the multicellularity of the host. Moreover, hosts usually harbor not only their own cells but also non-self cells. As the specialized compartments are highly interdependent, hosts usually provide common goods to all of their cells, for example, using the blood stream. This strategy is very effective, but susceptible to parasitism or the Tragedy of the Commons. To prevent this, hosts developed mechanisms for discrimination of damaged self cells as well as parasitic (or pathogenic) non-self cells from intact self cells and beneficial non-self cells and for restricting any non-self cells using the common goods. Beneficial non-self cells are hosted on the interface to the outside, e.g., the skin or the lumen of the gut, providing mutualistic protection and nutrition [28, 45, 110].

Protection inside the host is ensured by specialized cell lineages and their interactions, forming the immune system. In vertebrates, this system can be divided into innate immunity, detecting generic characteristics of non-self cells and tagging these cells, and adaptive immunity, which responds specifically to particular pathogens [91]. Both mechanisms rely on signals which, after interpretation by possibly several parts of the immune system, lead to an immune response. For example, vertebrates have developed a system in which each cell entering the blood stream is tagged non-specifically with complement factors like complement component 3b (C3b) [93]. Those tagging complement factors are inactivated specifically on intact self cells by inhibiting complement factors like factor H. If a phagocyte detects sufficient amounts of tagging complement factors on the surface of a cell, it will remove the cell. In contrast, if pathogens are able to remove the signal from their surfaces themselves, they could avoid attack.

To model HPI, it is thus important that the host has to classify the player types based on signals prior to interaction (e.g., self vs. non-self) to decide the optimal response strategy (e.g., attack or ’diplomat’ behavior [106]). This discrimination is prone to errors, since individuals imitating self-signals (mimicry) or preventing non-self-signals (crypsis) could dupe the host into using a non-desirable strategy. In HPI, both mimicry (e.g., resemblance of viruses to self-antigens) and crypsis (e.g., complement evasion by fungi and bacteria) occur. Molecular crypsis is achieved by several pathogenic fungi such as *C. albicans* and bacteria in that they are able to bind human factor H or other complement regulators on their surface. In this context, not only the evasion of pathogens is problematic but, because of the indistinguishability between self and non-self, also autoreactivity may arise [136].

Since mimicry and crypsis may occur whenever there is signal perception or communication involved, the type of mathematical model used has to be chosen depending on the concrete modeled system. Most commonly, theoretical descriptions distinguish between three entities: a model, a dupe, and a mimic [58]. Note the double meaning of the word ’model’ here: It refers to the mathematical description and to the template imitated by the mimic. The dupe learns the model based on the presence or absence of one or more characterizing signals. Due to noise in the perception system and diversity of the signal within the species, the perceived signal is usually modeled approximately using a Gaussian distribution [58, 133]. The closer the mimic resembles the model, the more the distributions will overlap, making it more difficult for the dupe to distinguish them. In agent-based models, signals could be inferred from explicit traits.

Given the signals, it is next important to define interactions between the three entities and the respective consequences on their fitness. This includes, for example, whether the model is attractive or should be avoided by the dupe. Also, the costs of erroneous classification as false positives or false negatives are very relevant. The classification task can then be modeled using any classifier, for example optimization of a classification threshold with respect to a fitness function or training of a neural network based on the previous interactions.

Perfect resemblance of the model signals by the mimic is possible in principle, no matter how sophisticated the classifier is, but may never be achieved. This is because the dupe and mimic constantly adapt and there is always an arms race in identifying and hiding, being attacked and evading [77, 81]. For example, in the case of complement evasion, most microorganisms are able to remove complement factors (which would identify them as pathogens) by utilizing the host-protecting complement factor H [137]. The host can make this adaption harder using polymorphous protection molecules, i.e., two different alleles of factor H and factor H-like molecules in addition.

In the case of adaptive immunity, pathogens could potentially mimic the serotype of self cells perfectly. Again, polymorphisms make adaption for pathogens more difficult. For example, the AB0 blood group polymorphism may protect against molecular mimicry (here on a population level), as pathogens mimicking type A antigens will not be able to survive in type B hosts [22]. Such mechanisms use the fact that pathogens usually transmit via several hosts, while host cells are not meant to migrate between hosts. On the other hand, the presence of mimicking pathogens will sensitize opposing types against each other, with great influence in transplantology. For example, a pathogen mimicking type A will induce anti-A antibodies in types B and 0.

**Fig. 2:**
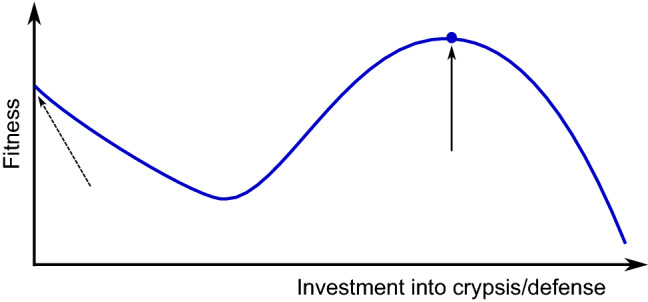
Qualitative dependence of fitness on the investment into crypsis or defense. The dashed arrow indicates the local maximum; solid arrow and blue dot: global maximum. The local maximum can be reached without additional investment. With a low amount of investment, the costs are higher than the benefit, which results in a decrease of fitness. Only a high investment is effective and leads to the global maximum. For further explanations, see section "Molecular mimicry and crypsis" and Lang et al. [77]

While mimicry has been intensely analyzed by mathematical modeling in the case of higher animals [58, 119], only a few modeling studies on camouflage by microorganisms have been published so far. The term ’molecular mimicry’ had been introduced by Damian in 1964 [22]. Recently, Lang et al. used signaling theory and ODEs to describe and analyze the attack decision by the host in the case of molecular crypsis [77]. That decision is non-trivial, because the two criteria of minimizing false negatives (foreign cells erroneously recognized as self) and minimizing false positives (own cells erroneously recognized as foreign) cannot be met simultaneously. The model identified relative pathogen abundance to be the predominant factor influencing successful molecular crypsis. If pathogen cells attain a quantitative advantage over host cells, even autoreactivity may occur. An alternative approach uses game theory [60]. It can nicely describe the trade-off between auto-immunity (e.g., age-dependent macula degeneration) and susceptibility to bacterial infections as well as the dimorphism of the factor H gene.

By plausibility arguments, the qualitative dependence of fitness on the investment into crypsis can easily be derived (Fig. 2). At low values of investment, fitness decreases practically linearly due to costs involved and because there is no benefit yet when the extent of crypsis is low. For example, a snake being purely black or purely red has achieved half of the coloring of poisonous snakes having black and red stripes but does not practically have any mimicry effect. Thus, there is a local maximum at zero investment. The global maximum is reached at a certain high investment if mimicry is efficient enough. A curve shape similar to that shown in Fig. 2 could indeed be obtained by numerical simulation of mimicry by *C. albicans* (Fig. 5 in [77]). In Fig. 2, the curve goes down beyond the global maximum based on the plausible assumption that an investment that is too high, takes away too much from the resources. It is an interesting open question how the global maximum could be attained in evolution as it is difficult to explain by small-scale mutations (micro-evolution). A possible solution is horizontal gene transfer, although this would not explain how the trait emerged in the first place.

## Spatial properties

There are two main approaches commonly used to model and simulate spatio-temporal dynamics: (i) a top-down equation-based modeling (EBM) approach and (ii) a bottom-up agent-based modeling (ABM) approach (also known as individual-based modeling). Each of these approaches has its own weaknesses and strengths depending on the questions to be answered and the hypotheses to be validated. The most widely used top-down approaches are differential equations-based: either ordinary (ODEs), partial (PDEs), stochastic (SDEs), or delay differential equations (DDEs). Another type is formed by discrete models such as difference equations.

ODE modeling is used, for example, to describe metabolic processes in pathogens [3]. The ODE or discrete models generally ignore the topology, while PDEs consider the spatial distribution to some extent (e.g., diffusion but not geometry of molecules). EBM does not deal with single entities but rather with describing populations by estimating the mean behavior at a macroscopic level [3, 62, 64, 69, 70, 88]. It uses a set of equations that are based on the relationships among observables. Solving these equations particularly in higher dimensions is based on numerical techniques, which reproduce the dynamics of variables (population densities, concentrations, etc.) over time. EBM has been widely employed to study the dynamics of bacteria [19, 88], fungi [79], viruses (for review, see [8, 12, 25, 52, 101, 139]), and has also been applied to other areas such as cell division mechanisms (e.g., [53, 63, 65–67, 129, 130]).

The bottom-up approach, including ABM or cellular automata (CA) such as the famous ’Game of life’ [42], works at a microscopic level [13, 14, 118]. Both ABM and CA are suitable methods to simulate the behavior of a system in a spatio-temporal manner. However, CA are grid-based and do not allow a free movement in space as the (usually) lattice-free ABM does [14, 21, 96, 118]. Thus, ABM can be considered as an extension or generalization of CA [26, 96]. In the unconventional ABM approach, individual independent agents are defined (such as fungal, bacterial, or human cells) with interaction rules [7, 13, 21, 44, 96, 118]. Individual interactions (usually between nearest neighbors) are simulated rather than global conditions [7, 21, 30, 114].

The behavior of the complete system arises from all local behaviors of the entities involved. This can lead to emergent properties of the system, which are not immediately seen from the local interactions. Even with simple rules, very complex spatio-temporal patterns can arise, as can already be seen in the ’Game of Life’. By ABM, the dynamics of different agents within a (complex) system can be analyzed and the impact of different initial settings can be investigated over time [7, 13, 21, 44, 118]. These bottom-up approaches are usually easier to implement than top-down approaches, because it is more comprehensible to describe the behavior of each individual agent than that of the whole system. Also, it can be adapted more flexibly to changing conditions and stochastic factors can easily be included in the rules [7, 13, 26, 96, 118]. A further advantage is that the results can be visualized nicely, for example, in colorful videos. Thus, ABM is a very powerful method to describe HPI and becomes increasingly important for the scientific community.

The power of ABM has its prize: Depending on the size of the system, ABM can be significantly more computationally expensive compared to EBM. Furthermore, ABM is often a stochastic rather than deterministic approach [7, 44, 96, 114, 118]. Consequently, it is not sufficient to run a simulation only once, but multiple simulations are required. The rules of the agents have to be realistic and can be gained from experiments, which become more and more available. However, this is not possible for all parameters and can lead to unknown values. Parameter estimation might be time consuming and may change the model outcome dramatically [26, 30, 118]. Taken together, ABM allows modeling with a higher spatial resolution over a longer time period compared to EBM, but it requires more detailed information about the system of interest and it is computationally more expensive. Without detailed knowledge, it might be too abstract for a certain application or may lack complex interaction mechanisms [96].

ABM has been applied across a wide range of disciplines including cell biology, population dynamics, epidemiology, and immunology [14, 21, 96, 117]. Many agent-based frameworks were developed (for review, see [1, 7, 15, 118]). Some famous examples are iDynoMics [78], MASON [82], NetLogo [132], and BSim [48, 85] which are user-friendly and can also be used by non-modelers. In addition, framework-independent implementations are used for ABM to simulate HPI [23, 102, 123]. NetLogo [21, 132] is one example for a modeling framework with its own programming language, with ‘turtles’ that represent agents and ‘patches’ that represent points in the simulation space. It can be applied easily to many different questions and has widely been utilized for HPI modeling [27, 89, 96, 112, 125]. NetLogo allows users to write their own extensions. However, it cannot incorporate formal rule-based languages such as BNGL (BioNetGen language) [10] or Kappa [16], nor molecular structure and geometry (for details, see [47, 49, 50, 54, 55, 71, 126]). In addition, it is challenging to handle very large network models or very low concentrations of agents with stochastic rules [68, 74, 75].

**Fig. 3:**
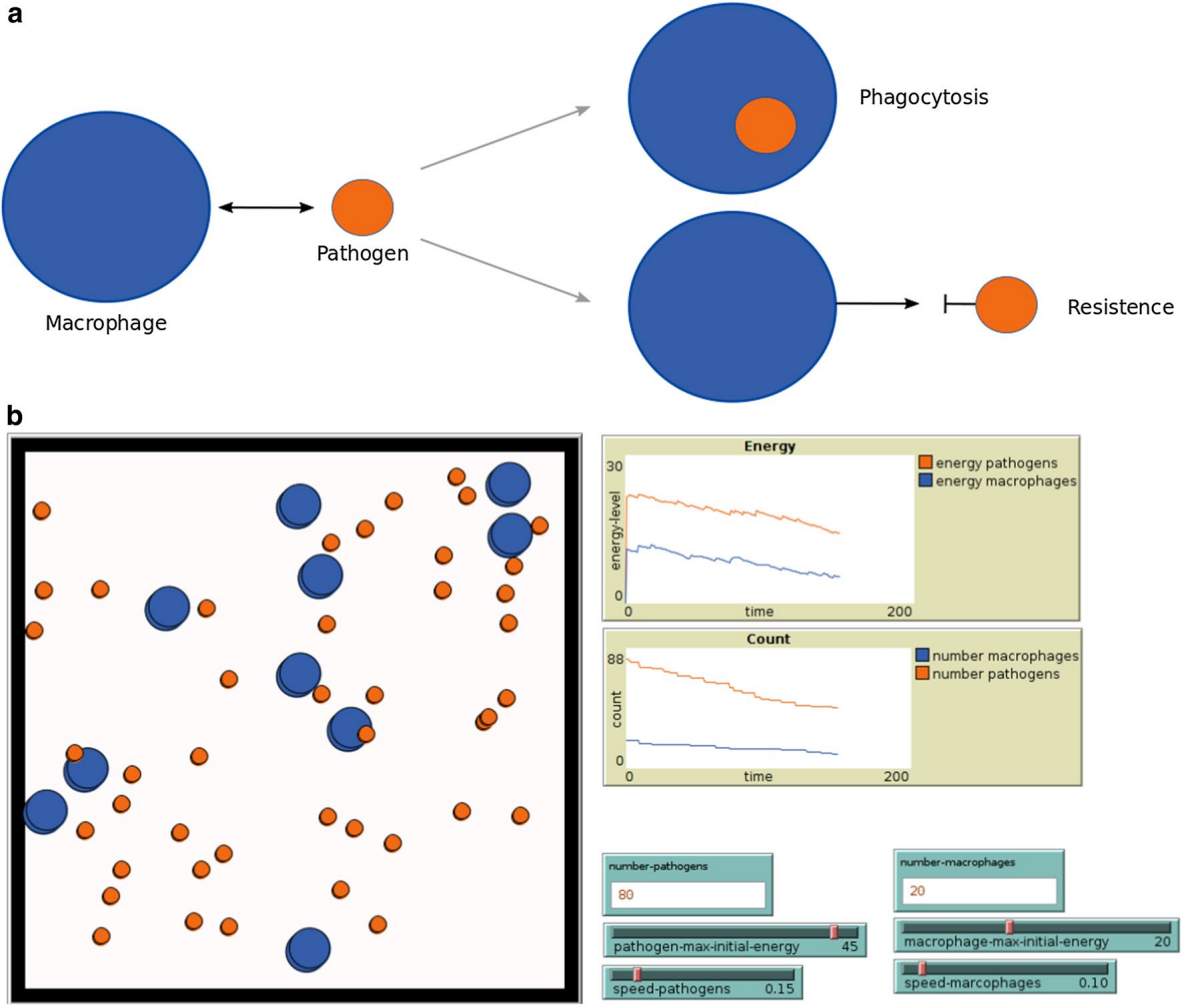
Toy example of HPI formalized as an agent-based model.** a** Individuals of macrophages (blue) phagocytose the pathogens (orange) when they are touching each other and the latter have too little energy to resist.** b** Screenshot of the simulation using NetLogo. During the simulation, the energy of macrophages and pathogens and the number of individuals are plotted

In the following, we provide a toy example using NetLogo to illustrate the ABM approach (see Fig. 3). Macrophages in interaction with a pathogenic species are modeled in a defined space. When macrophages recognize a pathogen, they try to phagocytose it. If the pathogen has too little energy to resist, it is taken up by the macrophage. In the implemented model (see Fig. 3), the user can define certain values for the number of individuals per species, its energy resources, and speed of movement. With these chosen parameter settings, the model can be initialized and run, and the movement of the agents can be visualized during the simulation. The time behavior of the energy level and number of species is monitored in different plots (see Fig. 3b). The simulated behavior can be extracted and processed or compared in further steps. In our example, it shows that the process of HPI is energy-consuming for both macrophages and pathogens. It depends on the number of macrophages how well pathogens can be phagocytosed.

Tokarski et al. implemented a related model using NetLogo, which shows that a communication between the immune cells (neutrophils in that case) is beneficial during HPI [125]. That model also indicated that hunting by neutrophils in clusters (like wolves in a wolfpack) is only beneficial if also the fungal pathogens occur in clusters. Otherwise, too much of the area remains uncovered by the neutrophils.

In another ABM, the movement of human alveolar macrophages searching *A. fumigatus* spores on the lung epithelium was modeled [102]. The model predicted that randomly migrating macrophages fail to find the spore before the start of germination, whereas guidance by chemotactic signals enables a safe and successful discovery of the pathogen in time (for an extended model, see [9, 103]). A further study about inhaled *A. fumigatus* spores considers a broader view of the lung tissue and immune cells [89]. This model focuses on the role of iron for both fungal and host cells, and shows that iron is a critical factor for fungal growth [89].

Bacterial infection has been investigated for *Pseudomonas aeruginosa* in the gut. It has been shown that an unbalanced gut flora can lead to increased virulence of *P. aeruginosa* and ABM was used to understand mechanisms of HPI [112, 123]. For example, Stern et al. used this approach to analyze the binding of the bacterium to the epithelium and suggest that integrin–laminin associations play an important role for bacterial virulence [123]. Furthermore, the infection of the intestinal tract by *Clostridium difficile* has been described by ABM to observe the efficiency of treatments against this bacterium [96]. The inflammation process in humans was in general formulated by ABM by Dong et al. They show that ABM is very suitable to describe complex systems, and model the host response and observe specific signaling, such as NF-$\kappa$B [27]. The NF-$\kappa$B pathway was investigated using ABM in other studies and reviewed by Williams et al. [134].

## Defense, counter defense, and counter–counter defense

In many parasitic interactions, the host organisms protect themselves by toxic compounds (defense chemicals) or other mechanisms such as mechanical barriers (e.g., skin), fever, pH changes, etc. Many of the pathogens, in turn, produce enzymes degrading the defense chemicals, which can be considered as a counter defense (a.k.a. evasion mechanisms). Examples are provided by the HPI between the human innate immune system and the pathogenic fungus *C. albicans* [30]. For example, immune cells produce various reactive oxygen species (ROS). *C. albicans* and many other pathogens, in turn, can detoxify the ROS by superoxide dismutases (SODs) [40]. All intracellular pathogens, like *Plasmodium* sp., also deflect defense to escape the humoral immunity of the host.

Interesting analogs can be found in plants. For example, the tomato plant produces tomatine to defend itself against the tomato-wilt fungus *Fusarium oxysporum* f. sp. *lycopersici* [94]. Tomatin is a glycoalkaloid with antimicrobial and insectidical properties. As a counter defense, the fungus produces tomatinase [94]. Another similar example is the oat plant which produces avenacin, a saponin which is mainly produced in the roots and provides defense against the fungal pathogen *Gaeumannomyces graminis* var. *avenae* by binding to sterols in the fungal cell membrane. The fungus responds by producing avenacinase, an enzyme that degrades avenacin [92].

Counter defenses represent the secondary phase of the HPI, i.e., they depend on the primary phase to be of any use at all. Thus, it might be risky to invest resources towards a robust counter defense without having a prior knowledge about the likelihood that the host will exhibit a formidable defense. Game theory and, more generally, operations research are powerful tools that can help to assess the costs and benefits of such interactions and the coevolution of host and parasite.

Clearly, the dependence of fitness on the investment into defense (or higher levels such as counter defense) is similar to that shown in Fig. 2. Again, a low extent of defense is practically useless, while it inflicts costs. For example, a toxin at low concentrations has (nearly) no effect. An interesting question is under which conditions it pays, during evolution, to establish a counter–counter defense rather than to intensify or widen an existing defense [30, 111].

Let us consider a game among a host and a pathogen where the cost of defense and counter defense is four units of resources. We assume that the pathogen has already played attack, and thus, the host starts the game with a damage of two units, i.e., the cost is twice as much as the damage. The pathogen has invaded the host successfully and, therefore, enjoys a reward of two units. The host can either choose to respond with a defense or choose to do nothing. The pathogen on the other hand can choose to do nothing or escalate the interaction to a further level by a counter defense. The payoffs for each of these strategies are summarized in Table 3.

**Table 3:**
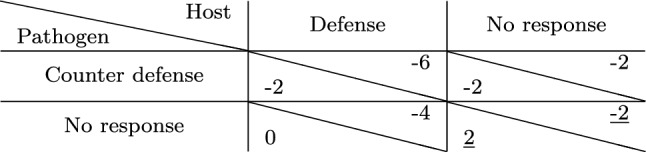
Payoff matrix of a game between host and parasite with defense and counter defense. Payoffs are such that the cost of defense is twice as much as the damage. The Nash equilibrium is underlined

In the scenario where both players choose the non-peaceful strategy, i.e., the host chooses to defend itself and the pathogen responds with a counter defense, both incur a cost of four units. In the absence of defense, counter defense only costs the pathogen four units of resources. In a scenario where the pathogen does not respond to a defense of the host, the host incurs a cost of four units. When both players choose to show no response, the host has a damage of two units from the attack, while the pathogen, having invaded the host, enjoys a reward of two units as per assumption. Clearly, choosing the peaceful ‘no response’ strategy by both players seems the least costly choice. This is also the Nash equilibrium, since neither player has an incentive to change strategy unilaterally.

Let us now consider a scenario where the cost is only one unit, i.e., the damage is twice as expensive as the cost. The resulting payoff matrix is depicted in Table 4 and shows that the host has an advantage in playing defense, since it gives a better payoff than when none of the players show any response. However, the pathogen has an incentive to respond with a counter defense, implying that the host obtains the worst payoff possible. Thus, the host is likely to choose no response again, leading to a cycle. There is no pure Nash equilibrium for the game.

**Table 4:**
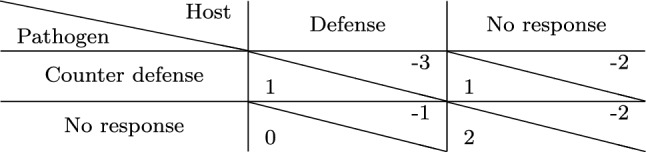
Payoff matrix of the defense/ counter defense game as in Table 3 but with the cost being half of the damage. This game possesses no pure Nash equilibrium

The shuffle through the strategies could occur, for example, through gain and loss of function in various genes responsible for (counter)defense. Moreover, it is worth noting that the above approach is based on a discretization to two strategies only on each side. In reality, the amount of the defense chemical and the counter defense (e.g., degrading enzyme) can be chosen in a continuous way. A mixed Nash equilibrium could then mean that a certain percentage of the full amount is produced.

By the traditional approach of game theory used here, it cannot be decided whether a self-sustained oscillation in time between the two choices arises or the strategies are chosen with a certain probabilities in a stochastic way as in the famous rock–scissors–paper game [46] or only part of the defense/counter defense is realized. One way of answering that question is using ODEs, as was used for modeling the rock–scissors–paper game among bacteria [88].

## Conclusion and outlook

In recent years, mathematical and computational approaches have successfully been applied to study HPI from various angles. In synergy with experimental work, in silico models of HPI led to a better understanding of virulence factors and their evolution, a disentanglement of the complex immune response and suggestions for novel or improved antibiotic treatments.

For the future, we see several trends impacting the modeling of HPI. First, the implementation and simulation of models like agent-based and dynamic optimization models is becoming more and more practicable for non-experts by user-friendly tools such as NetLogo or AMIGO2 (toolbox for dynamic modeling and optimization [4]). The second major trend is the availability and integration of vast biological data from omics approaches and imaging. These data allow dynamic and spatial modeling to be highly quantitative and to reach a better predictive power. A similar goal is pursued by multi-scale modeling, which is an additional important trend in models of HPI.

It is often useful to validate mathematical models by alternative modeling methods and frameworks [91], to verify that the results are not an artifact of the particular framework used. As discussed in detail by Schleicher et al. [109], the combination and integration of different approaches is valuable to exploit the large amount of biological data and complex networks of HPI. A combined approach often improves the applicability and value of mathematical models, as pointed out in Section "Defense, counter defense and counter-counter defense" with respect to game theory and ODEs [9, 19, 29, 56, 88]. Moreover, game-theoretical approaches can be combined with ABM, dynamic optimization [29], or in the form of games on grids or graphs [107].

**Fig. 4:**
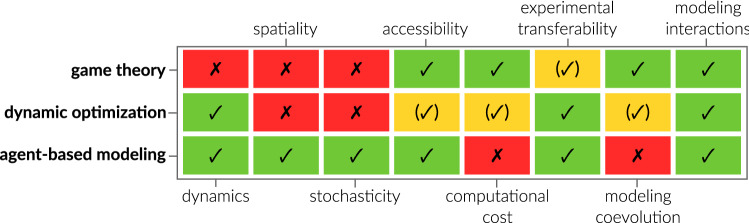
Comparison of methods presented and discussed in detail in this review. Classification (fully ✓, partially (✓), and not capable ✗) is based on the original method without special extensions and according to its application in HPI modeling as well as the authors’ experience

Associated with numerous computational approaches, the challenge is to choose the appropriate modeling approach. While there is no definitive answer to the question which modeling approach and method is best to model an HPI, one can base the decision on general method characteristics (see Fig. 4). Important distinctive features of the mainly presented methods from game theory, dynamic optimization, and ABM are their capabilities to resolve system dynamics, spatiality, and stochasticity. This means, for example, that one should consider modeling an HPI by ABM if interaction partners occur in low numbers (stochasticity) or diffusion of signaling chemicals is important (spatiality). While classical game theory does not resolve dynamics, spatiality, or stochasticity per se, it inherently can model coevolution, since interaction strategies are optimized simultaneously and not independently (as in dynamic optimization) or are fixed (as in ABM). Furthermore, the analysis does not require high computational power like ABM due to its stochastic trait. However, a major drawback is the higher abstraction level of biological systems in game theory, which complicates experimental validation of results in comparison to dynamic optimization and ABM.

As a major strength of all three discussed approaches, we see their accessibility to non-expert users and most importantly their ability to model HPI. These HPI are either directly implemented as interaction events in ABM, interaction kinetics in dynamic optimization, or strategies in game theory.

Prospectively, we see the challenge that models of HPI will be more complex in the future due to the amount of experimental data. This is in contrast to the requirement that models should be comprehensible and minimal, so that a trade-off has to be found. Despite these challenges, efforts in mathematical and computational modeling pay off. For example, spatial modeling disclosed that immune cells benefit from chemotaxic signals to effectively clear pathogens [102, 125]. These findings now require more experimental work for elucidation. Furthermore, epidemiological modeling of malaria shows the value of combining spatial and dynamic modeling to explore strategies eliminating infectious diseases [32]. Further important examples have been discussed throughout this review. This shows the important value of mathematical modeling in understanding host–pathogen interactions.

Also in the future, modeling of HPI will be an important complement to experimental work to simplify scientific procedures, to reduce (animal) experiments, and to generate hypotheses. We assume that the accessibility of modeling approaches is key to make them applicable for more scientists and recommend the presented approaches which are usable by non-experts. Furthermore, dynamics and spatial observations may have a higher impact over time to describe more complex behavior. This can give rise to new (sub-)approaches to model HPI.

Due to increasing computational power, the limitation of computation (e.g., in ABM and dynamic optimization) will decrease and enable a higher workload and the implementation of large-scale models. Nevertheless, the key in future modeling will be the integration of experimental data and a close collaboration of computational modelers and biological as well as medical experts. This ensures validity of models and decipherment of HPI.
